# Intralogistik im Zeitalter des digitalen Wandels: Arbeitsanforderungen und psychische Beanspruchung in der Kommissionierung am Beispiel eines Unternehmens aus dem E-Commerce-Bereich

**DOI:** 10.1007/s41449-021-00285-4

**Published:** 2021-10-13

**Authors:** Gerhard Rinkenauer, Julian Elias Reiser, Johanna Renker, Veronika Kretschmer

**Affiliations:** 1 Leibniz-Institut für Arbeitsforschung an der TU Dortmund (IfADo), Ardeystraße 67, 44139 Dortmund, Deutschland; 2 Technologieberatungsstelle beim DGB NRW e. V. (TBS NRW), Dortmund, Deutschland; 3 Fraunhofer-Institut für Materialfluss und Logistik IML, Joseph-von-Fraunhofer-Str. 2–4, 44227 Dortmund, Deutschland

**Keywords:** Anforderungen, Psychische Beanspruchung, Ressourcen, Mitarbeiterbefragung, Kommissionierung, Logistik, Job demands, Mental strain, Resources, Employee survey, Order picking, Logistics

## Abstract

Der E‑Commerce-Bereich erfährt aufgrund der Digitalisierung einen kontinuierlichen Aufschwung in der Logistik. Der Mensch bleibt vor dem Hintergrund der mehrheitlich manuell ausgeführten Tätigkeiten eine entscheidende Ressource im Lager, die es, mit besonderem Blick auf den demografischen Wandel, zu halten und zu integrieren gilt. Im Beitrag werden die Befragungsergebnisse einer Feldstudie mit dem Fokus auf den Bereich der Kommissionierung, die bei einem großen Versandhändler durchgeführt wurde, exemplarisch beschrieben und mit Befragungsergebnissen von anderen Betrieben verglichen. In Anlehnung an das Anforderungs-Ressourcen-Modell werden neben den Zielgrößen Arbeitsfähigkeit, Arbeitszufriedenheit und Gesundheit, Stressoren, Herausforderungen und Ressourcen beleuchtet. Die Ergebnisse deuten darauf hin, dass sich Stressoren und Arbeitsressourcen in etwa ausgleichen, auch wenn sich z. B. Arbeitszufriedenheit und Gesundheit lediglich auf einem mittleren Niveau befinden. Vor allem die Autonomie der Beschäftigten birgt noch Handlungsbedarf. Die digitale Transformation wird als Chance gesehen, den Handlungs- und Entscheidungsspielraum zu erweitern.

*Praktische Relevanz:* Die operative Logistik birgt aufgrund der manuell geprägten Tätigkeiten und der vorgegebenen Prozesse eine Vielzahl an Arbeitsanforderungen, die zu psychischer Fehlbeanspruchungen und langfristig zu Störungen oder Erkrankungen führen können. Zur Kompensation der Stressoren ist die Erweiterung von arbeitsbedingten Ressourcen notwendig. Die Digitalisierung wird als Chance gesehen, diese auszubauen.

## Abstract (en)

The e‑commerce sector is experiencing a continuous boom in logistics due to digitization. Along with mostly manual activities, people remain a decisive resource in the warehouse, which must be retained and integrated with a particular view to demographic change. In the article, the survey results of a field study with focus on the area of order picking, which was conducted at a large mail-order company, are described as an example and compared with survey results from other companies. Based on the job demand resources model, stressors, challenges and resources are examined in addition to the target variables of work ability, job satisfaction and health. The results indicate that stressors and work resources roughly keep the balance, even if, for example, job satisfaction and health are only at a medium level. Above all, there is still a need of action regarding autonomy of employees. The digital transformation is seen as an opportunity to expand the scope for action and decision-making.

*Practical Relevance:* Due to the manual nature of the activities and the predefined processes, operational logistics involves a large number of work demands that can lead to mental strain and, in the long term, to disorders or illnesses. The expansion of work-related resources is necessary to compensate for the stressors. Digitization is seen as an opportunity to expand these resources.

## Einleitung

### Logistik im Wandel

Die Logistikwirtschaft in Deutschland ist im Jahr 2019 erneut gewachsen, z. B. bzgl. des Gesamtvolumens, der Beschäftigungszahlen sowie der zu bewältigenden Gütergesamtmenge. Insbesondere der E‑Commerce-Bereich, der den Verkauf von physischen Gütern, u. a. der Segmente Elektronik und Medien, Fashion, Möbel und Haushalt, an private Endnutzer über einen digitalen Kanal (B2C) umfasst, erfährt einen großen Aufschwung. Gerade auch vor dem Hintergrund des veränderten Einkaufsverhaltens von Offline- hin zu Online-Käufen im Zuge aktueller Trends und der COVID-19-Pandemie wird der E‑Commerce-Sektor als ein Impulsgeber für die Logistikwirtschaft gesehen. Prognosen gehen dahin, dass der E‑Commerce-Markt auch zukünftig stark wachsen wird. Generell findet in der Logistik eine starke Durchdringung der Digitalisierung statt, um auf Flexibilitätsanforderungen der modernen Gesellschaft reagieren zu können.

**Figure 1:**
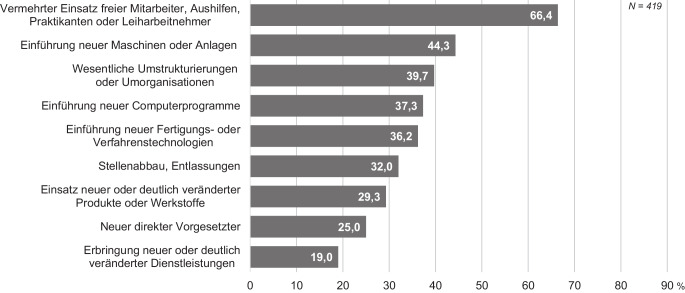

Diese zunehmende Digitalisierung und die damit einhergehenden Veränderungen im unmittelbaren Arbeitsumfeld bewirken insbesondere in der operativen Logistik neue Herausforderungen zukünftiger Arbeitsumgebungen (Abb. 1). Potenzielle Nachteile bestehen darin, dass Beschäftigte zunehmend mit neuen oder veränderten Belastungen und Beanspruchungen konfrontiert sind, wie zum Beispiel vermehrtem Stressempfinden durch die aus der Digitalisierung entstandenen Überwachungsmöglichkeiten und Leistungskontrolle (Khanchel 2019). Die Arbeitsabläufe werden aufgrund zunehmender Informations- und Kommunikationsanforderungen komplexer und gehen mit einem größeren Zeitdruck einher. Aufgrund der zum Teil disruptiven Veränderungen von Arbeitsumgebungen ist es erforderlich, potenzielle psychische Fehlbeanspruchungen bereits im Vorfeld über unterschiedliche methodische Zugänge zu erkennen und entsprechende Handlungs- und Gestaltungsempfehlungen zur Verfügung zu stellen (z. B. Certa und Schröder 2020). Während im Bereich der Kommissionierung physische Fehlbeanspruchung inzwischen im Fokus der Forschung steht (z. B. Grosse et al. 2017) und auch psychische Fehlbeanspruchung zumindest im Kontext theoretischer Konzepte immer häufiger diskutiert wird (z. B. Winkelhaus et al. 2021), gibt es bisher kaum empirische Untersuchungen zur psychischen Beanspruchung.

### Theoretische Grundlagen

#### Theoretischer Rahmen

Als Leitfaden und theoretischer Rahmen unserer Studien im Kontext psychischer Fehlbeanspruchung dient das Anforderungs-Ressourcen-Modell (JD-R-Modell: job demands-resources model, Demerouti et al. 2001; Schaufeli und Taris 2014). In den letzten Jahren hat sich das JD-R-Modell zu einem der einflussreichsten Stress- und Motivationsmodelle in der Arbeits- und Organisationspsychologie entwickelt. Grundannahme des JD-R-Modells ist, dass alle psychosozialen Arbeitsmerkmale in Anforderungen und Ressourcen kategorisiert werden können (Demerouti et al. 2001; Schaufeli und Bakker 2004). Demerouti et al. (2001) definieren Arbeitsanforderungen als physische, psychische, soziale oder organisatorische Aspekte einer Arbeit, welche anhaltende physische und/oder psychische Anstrengung erfordern (z. B. Arbeitsbelastung, Rollenkonflikte). Bakker und Demerouti (2007) gehen davon aus, dass Anforderungen nicht unbedingt negativ für das Beanspruchungserleben sind, sondern erst dann zu Stressoren werden, wenn deren Erfüllung hohe Anstrengungen erfordert und man sich von diesen Anstrengungen nicht genügend erholen kann.

Arbeitsanforderungen gelten als Stressoren, wenn sie als Hindernisse auf dem Weg zur Zielerreichung und Bedürfnisbefriedigung wahrgenommen werden und dadurch die Arbeitsfähigkeit und Gesundheit gefährden. Arbeitsanforderungen werden hingegen als Herausforderungen bewertet, wenn sie der Zielerreichung und Bedürfnisbefriedigung zuträglich sind und deren Kosten deshalb in Kauf genommen werden (Berhelson et al. 2018). Arbeitsanforderungen können damit einerseits die Arbeitsfähigkeit und Gesundheit gefährden (z. B. durch Verausgabung), dienen aber zugleich auch der Motivation und damit dem Wohlbefinden. Beispielsweise wird Arbeitsintensität (hohes Arbeitspensum, Zeitdruck) als Herausforderung wahrgenommen, wenn sie beherrschbar erscheint und als Stressor, sobald sie nicht mehr kontrolliert werden kann (Widmer et al. 2012).

Arbeitsressourcen adressieren Aspekte der Arbeit, die Arbeitsbelastungen und die damit verbundenen physiologischen und psychologischen Kosten reduzieren können, funktional für das Erreichen von Arbeitszielen sind und darüber hinaus persönliches Wachstum, Lernen und Entwicklung (Bakker und Demerouti 2007). Arbeitsressourcen gehen aus der Arbeitsaufgabe selbst hervor (z. B. Ganzheitlichkeit, Anforderungsvielfalt, Bedeutsamkeit), sie entspringen aus der Aufgabenorganisation (z. B. Partizipation, Rollenklarheit, Entscheidungsfreiheit) oder ergeben sich aus dem sozialen Umfeld (Unterstützung von Kollegen und Vorgesetzten, kollegiale Atmosphäre). Nicht zuletzt kann die Organisation als Ganzes bezüglich Bezahlung, Karrieremöglichkeiten oder Arbeitsplatzsicherheit als Ressource gesehen werden.

Nach dem JD-R-Modell können Mitarbeiter auch ihre persönlichen Ressourcen nutzen, um mit den Arbeitsanforderungen umzugehen (Bakker und de Vries 2021). Persönliche Ressourcen beziehen sich auf die Selbsteinschätzung, wie viel Kontrolle eine Person über die Arbeitsumgebung hat. Genau wie die Arbeitsressourcen sind persönliche Ressourcen wie Optimismus, Selbstwirksamkeit und Resilienz motivierend, weil sie den Mitarbeitern helfen, ihre arbeitsbezogenen Ziele zu erreichen.

Generell geht das Modell davon aus, dass hohe Arbeitsanforderungen einen Prozess der gesundheitlichen Beeinträchtigung auslösen können (Überlastung, Burnout und Krankheit), während hohe Arbeitsressourcen energetisierend sind, indem sie Motivationsprozesse in Gang setzen, die zu einer positiven Einstellung gegenüber der Arbeit und zu positivem Verhalten führen und Belastungssymptome reduzieren können (Bakker und Demerouti 2007; Berthelsen et al. 2018).

#### Bezug zur Intralogistik

Das JD-R-Modell wird bisher eher selten in der Logistik angewendet (z. B. Semeijn et al. 2019; Certa und Schröder 2020). Dies kann darin begründet sein, dass in diesem Bereich bisher, wie oben erwähnt, nur wenige Studien bezüglich psychischer Beanspruchung durchgeführt wurden. Certa und Schröder (2020) verwendeten beispielsweise das JD-R-Modell als theoretischen Rahmen, um veränderte Arbeitsanforderungen durch die zunehmende Digitalisierung in der Intralogistik und im Zustellbereich empirisch zu untersuchen. Zentral war die Frage, in welchem Zusammenhang die Arbeitsfähigkeit der Mitarbeiter mit den Arbeitsbedingungen steht. Als Grundlage für die Untersuchungen diente eine umfangreiche Arbeitszeitbefragung der BAuA. Die Analysen erfolgten im Kontext von Regressionsmodellen, wobei als zentrale, abhängige Variable die subjektive Bewertung der eigenen Arbeitsfähigkeit verwendet wurde. Als unabhängige Variablen dienten Stressoren, Herausforderungen, organisationale und individuelle Ressourcen sowie soziodemografische Merkmale.

Die Ergebnisse der Studie von Certa und Schröder (2020) zeigen insbesondere hinsichtlich psychischer Fehlbeanspruchung, dass im Vergleich zu anderen Erwerbstätigen beide Berufsgruppen ihre Arbeitsfähigkeit schlechter als der Durchschnitt beurteilen, sie sind im Wesentlichen häufiger physischen Stressoren ausgesetzt, haben geringere organisationale Ressourcen, wie Anerkennung sowie soziale Unterstützung durch Kollegen oder Führungskräfte oder beruflichen Entscheidungsspielraum, sowie ein geringeres Bildungsniveau als individuelle Ressource. Schnelles Arbeiten kann als Herausforderung gewertet werden, da es für beide Berufsgruppen positiv mit der Arbeitsfähigkeit assoziiert ist. Erst wenn ein starker Termin und Leistungsdruck wahrgenommen wird, besteht Stress, der sich in einer geringeren Arbeitsfähigkeit ausdrückt. Die Nutzung von Informations- und Kommunikationstechnologien wird in den Lagerwirtschaftsberufen als Stressor und nicht als Herausforderung identifiziert. Hingegen kommen in der Lagerwirtschaft die Ressourcen einer sozialen Unterstützung durch Kollegen sowie eines Klimas der Rollenklarheit den Beschäftigten zugute. Als individuelle Ressource gehen die Selbstwirksamkeit aber auch die Besetzung einer Führungsposition mit einer erhöhten Arbeitsfähigkeit einher.

#### Konzept der vorliegenden Feldstudie

**Figure 2:**
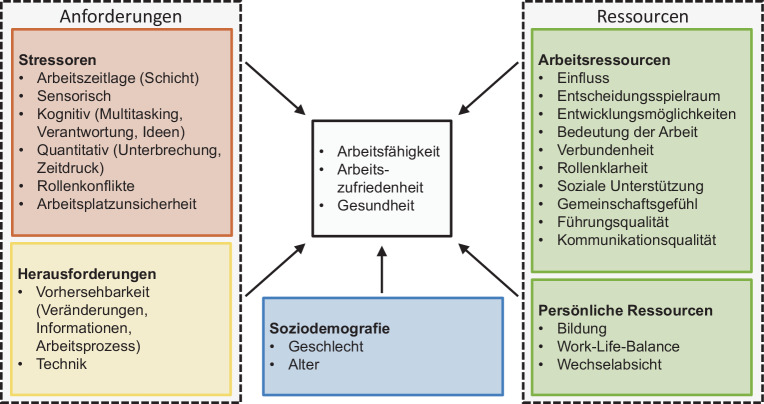

In unserer Studie orientieren wir uns an dem Anforderungs-Ressourcen-Modell von Certa und Schröder (2020) und betrachten bei unseren Erhebungen relevante Stressoren, Herausforderungen und Ressourcen, sowie soziodemografische Variablen (Abb. 2), um die Arbeitsfähigkeit, Arbeitszufriedenheit und Gesundheit im Bereich der Kommissionierung auf einer deskriptiven Basis beurteilen zu können. Als Messinstrument wurde ein, im Wesentlichen auf dem Copenhagen Psychosocial Questionnaire (COPSOQ, Nübling et al. 2005) basierend, zusammengestellter Fragebogen verwendet, und die entsprechenden Dimensionen des COPSOQ dem JD-R-Modell zugeordnet. Das Instrument eignet sich als Screening-Tool für die betriebliche Praxis, kann aber auch zu Forschungs- und Evaluationszwecken eingesetzt werden (Nübling et al. 2005). Berthelsen et al. (2018) haben gezeigt, dass die Konstruktvalidität des COPSOQ-Instruments prinzipiell in das JD-R-Rahmenwerk integriert werden kann.

Bedingt durch den demografischen Wandel wurden junge und alte Beschäftigte des in diesem Beitrag untersuchten Versandhändlers gegenübergestellt, um Erkenntnisse über die Auswirkung arbeitsrelevanter Faktoren auf die Arbeitszufriedenheit und Gesundheit zu gewinnen.

## Methodik

### Durchführung der Mitarbeiterbefragung

Die in diesem Beitrag vorgestellte Feldstudie wurde im Rahmen des Forschungsthemas „Angewandte kognitive Ergonomie“ der Initiative der Fraunhofer-Gesellschaft „Leistungszentrum Logistik & IT“, die mit Mitteln der Fraunhofer-Gesellschaft und des Landes Nordrhein-Westfalen gefördert wird, durchgeführt. In neun deutschen Logistiklägern verschiedener Branchen wurden Paper-Pencil-Mitarbeiterbefragungen durchgeführt, um die Arbeitsfähigkeit, Arbeitszufriedenheit und Gesundheit von Mitarbeiter/-innen unter Berücksichtigung der Anforderungen und Ressourcen in der Kommissionierung zu untersuchen. Es werden die Ergebnisse einer Befragung im E‑Commerce-Bereich eines großen Unternehmens exemplarisch beschrieben. In dem Kommissionierbereich des Versandhändlers wird in Wechselschicht (Früh- und Spätschicht) gearbeitet. Insgesamt nahmen *N* = 49 Beschäftigte an der 1 ¼-stündigen Mitarbeiterbefragung teil.

In dem untersuchten Unternehmen wird ein beleggebundenes Kommissionierverfahren eingesetzt, das die Mitarbeiter/-innen dabei unterstützt, den Kommissionierauftrag zusammenzustellen. Die Kommissioniertechnik basiert auf einer papierbasierten Informationsbereitstellung in Form von Kommissionier-Etiketten (sog. Warenbegleitscheine). Diese enthalten alle für die Kommissioniertätigkeit relevanten Informationen, wie z. B. Artikelname, Lagerplatz, benötigte Entnahmemenge, Materialnummer und Chargennummer.

Der für die Feldstudie konzipierte Fragebogen basiert im Wesentlichen auf dem COPSOQ (Abschn. 1.2.3). Die ausgewählten Skalen und Einzelitems wurden mittels 5‑stufiger Antwortskalen erfasst und auf einen Wertebereich von 0 bis 100 transformiert (0 = minimale Ausprägung – „nie/fast nie“, „trifft gar nicht zu“ bzw. „in sehr geringem Maß“; 100 = maximale Ausprägung – „immer“, „trifft voll zu“ bzw. „in sehr hohem Maß“) (Nübling et al. 2005).

### Statistische Analyse

Für eine explorative Gegenüberstellung der Fragebogendaten, des hier vorgestellten Kommisionierbereichs des Versandhändlers und der anderen in der Befragung erfassten Unternehmen, wurden t‑Tests für unabhängige Stichproben durchgeführt. Für kleine Stichproben sind hierzu Bootstrap-Verfahren (1000 Iterationen pro Bootstrap) zur Schätzung der Konfidenzintervalle besonders geeignet. Mit diesem Verfahren wurden alle in diesem Artikel tabellarisch dargestellten Skalen miteinbezogen. Aufgrund des hohen mittleren Alters der Belegschaft des dargestellten Unternehmens, wurde das gleiche Verfahren für die Gegenüberstellung der jüngeren (<45 Jahre) und älteren Belegschaft (≥45 Jahre) innerhalb des Unternehmens angewandt, um mögliche Altersunterschiede zu untersuchen.

Statistische Berechnungen wurden mit RStudio Version 15.6.0 (RStudio Team 2020, Wollschläger 2017) durchgeführt. Alle Ergebnisse wurden in den jeweiligen Tabellen dargestellt. Die Tabelleneinträge sind wie folgt zu deuten: 0 = kein signifikanter Unterschied, − = nicht geprüft; erster Eintrag mit > oder < bedeutet, dass die Stichprobe des Versandhändlers einen signifikant größeren (>) oder kleineren Mittelwert (<) im Vergleich zur restlichen Stichprobe zeigt; zweiter Eintrag mit > oder < bedeutet, dass der jüngere Teil der Stichprobe des Versandhändlers einen signifikant größeren (>) oder kleineren Mittelwert (<) als der ältere Teil der Stichprobe zeigt. Die einzelnen, ausführlichen Ergebnisse, inklusive der Teststatistiken, sind im Fließtext angeführt.

## Ergebnisse der Feldstudie am Beispiel eines Versandhändlers

Nachfolgend werden ausgewählte deskriptive Ergebnisse der Themenbereiche entsprechend Abb. 2 vorgestellt.

### Soziodemografie

Die Stichprobe der *N* = 49 befragten Mitarbeitenden der operativen Kommissionierung besteht mehrheitlich aus Frauen (87,8 %) mit einem Durchschnittsalter von 48,9 Jahren (*SD* = 9,4) und arbeitet in Vollzeit (71,4 % vs. 28,6 % Teilzeit mit einer wöchentlichen Arbeitszeit von 15 bis 34 h). Der Großteil der Beschäftigten ist 45 Jahre alt und älter (25–34 Jahre: 10,4 %, 35–44 Jahre: 14,6 %, 45–54 Jahre: 45,8 %, >54 Jahre: 29,2 %). Um einen Vergleich des hier näher beschriebenen Versandhändlers und den übrigen Unternehmen zu ermöglichen, wurden die Referenzbetriebe (*N*_Referenz_ = 158) ebenfalls auf ihre soziodemographischen Merkmale geprüft. Das Alter in den Referenzbetrieben ist auf einem vergleichbaren Niveau (*M*_Referenz_ = 41,2, SD_Referenz_ = 10,7). Das Geschlechterverhältnis (Weiblich_Referenz_ = 34 %) weicht stark vom untersuchten Betrieb ab.

### Anforderungen

Entsprechend des vorgestellten Anforderungs-Ressourcen-Modells (Abb. 2) wird den Anforderungen der Beschäftigten in der Kommissionierung ein Einfluss auf die Zielgrößen Arbeitsfähigkeit, Arbeitszufriedenheit und Gesundheit zugeschrieben. Die Anforderungen lassen sich in Stressoren und Herausforderungen unterteilen. Nachfolgend werden zunächst sowohl deskriptiv als auch inferenzstatistisch die Auswertungen der Arbeitsstressoren und im Anschluss die der Herausforderungen bei der Arbeit vorgestellt und beschrieben (Tab. 1).

**Table 1:** 

Einzelitems/Skalen	Herkunft	*N* Items	*M* ± SD	Sign. Unterschiede
*Stressoren*				
Sensorische Anforderungen	COPSOQ	5	95,8 ± 7,4	>;0
Kognitive Anforderungen	COPSOQ	8	42,9 ± 16,9	<;<
Quantitative Anforderungen	COPSOQ	4	60,8 ± 15,4	0;0
Rollenkonflikte	COPSOQ	4	39,5 ± 21,0	−;0
Arbeitsplatzunsicherheit	COPSOQ	4	47,8 ± 25,7	0;0
*Herausforderungen*				
Vorhersehbarkeit	COPSOQ	2	51,0 ± 20,6	−;0
Technik	ISONORM	7	1,0 ± 1,0	>;−

#### Stressoren

Zu den abgefragten Stressoren zählen bspw. Schichtarbeit oder sensorische, kognitive und quantitative Anforderungen. Die Ergebnisse zeigen, dass sich 44,9 % der Personen in der Kommissionierung, und damit fast die Hälfte, durch das vorherrschende Schichtarbeitszeitmodell (im Wechsel Früh- und Spätschicht) belastet fühlt. Die Mehrheit der Beschäftigten im Lager gibt an, dass sie etwa zu gleichen Teilen geistig und körperlich tätig ist (83,7 %). Demgegenüber schätzen 16,3 % der Befragten ihren Tätigkeitsinhalt als vorwiegend körperlich geprägt ein (Tuomi et al. 1998).

**Figure 3:**
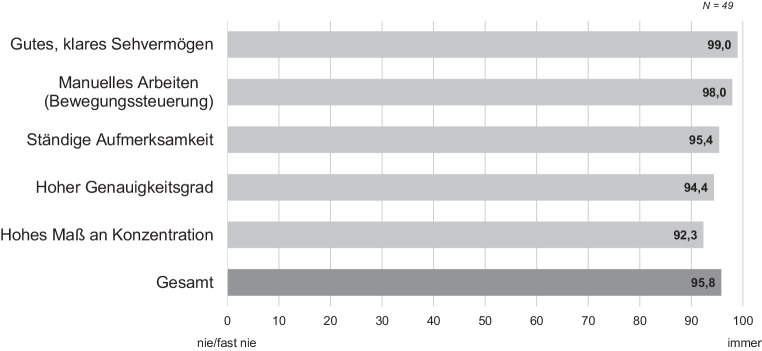

Weiterhin zeigt die Auswertung, dass Anforderungen an die Sensorik des Menschen am häufigsten vorliegen (Tab. 1). Die Ergebnisse deuten darauf hin, dass die Befragten beim Kommissionieren fast immer äußere Reize sensorisch, insbesondere visuell und haptisch, verarbeiten müssen (Abb. 3). Die Kommissioniertätigkeit verlangt neben einem guten, klaren Sehvermögen und manueller Bewegungssteuerung außerdem in einem hohen Maß konzentriertes, aufmerksames und genaues Arbeiten. Im Vergleich zu Beschäftigten anderer Unternehmen fällt die sensorische Arbeitsanforderung signifikant höher aus (*M*_Betrieb_ = 95,8, *M*_Referenz_ = 86,6; CI_Bootstrap_ = (6,54, 13,35); *t*(201) = 3,92, *p* < 0,001). Innerhalb des Unternehmens unterscheiden sich sensorische Anforderungen interessanterweise nicht für jüngere und ältere Mitarbeiter/-innen (*p* = 0,39).

**Figure 4:**
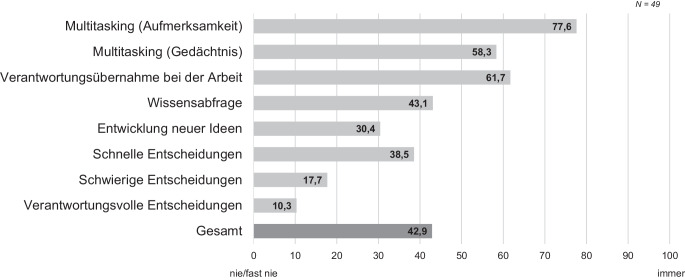

Zu den häufig auftretenden kognitiven Anforderungen (Tab. 1) gehören Multitasking, das Treffen von Entscheidungen, Verantwortungsübernahme bei der Arbeit sowie das Abrufen von Wissen. Die Befragten in der Kommissionierung berichten, dass sie bei der Arbeit am häufigsten mit Multitasking und der Übernahme von Verantwortung als Stressoren umgehen müssen (Abb. 4).

Im Vergleich zu anderen Unternehmen zeigt sich, dass sich Mitarbeitende des untersuchten Betriebs weniger kognitiv gefordert fühlen (*M*_Betrieb_ = 42,9, *M*_Referenz_ = 53,7; CI_Bootstrap_ = (−16,51, −5,57); *t*(202) = −3,52, *p* < 0,001). Auch innerhalb des Versandhändlers unterscheiden sich kognitive Anforderungen signifikant, jüngere Mitarbeiter/-innen empfinden weniger kognitive Anforderungen (*M*_jung_ = 33,3, *M*_alt_ = 49,0; CI_Bootstrap_ = (−24,48, −7,53); *t*(46) = −3,58, *p* < 0,001).

**Figure 5:**
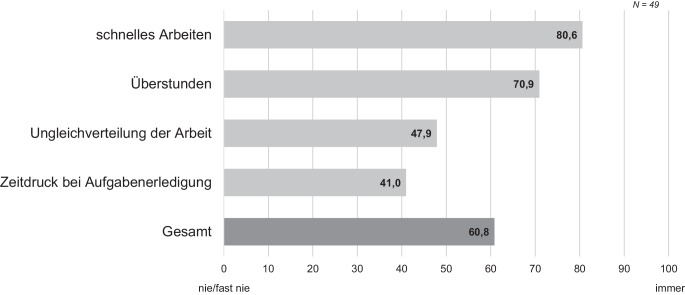

Hinsichtlich der quantitativen Anforderungen zeigt sich, dass die Lagerarbeitenden oft Anforderungen bewältigen, die sich aus der Arbeitsmenge, -zeit und -geschwindigkeit ergeben (Tab. 1). Die Tätigkeit in der Kommissionierung ist durch eine hohe Arbeitsintensität gekennzeichnet (Abb. 5). Dabei kommt es gelegentlich zu einer Ungleichverteilung der Arbeit und einem empfundenen Zeitdruck (Abb. 5). Bezüglich der quantitativen Arbeitsanforderungen zeigen sich keine besonderen Muster bei dem untersuchten Versandhändler (*M*_Betrieb_ = 60,8, *M*_Referenz_ = 54,2; CI_Bootstrap_ = (1,18, 11,59); *t*(204) = 2,30, *p* < 0,01) oder zwischen Jung und Alt (*M*_jung_ = 55,0, *M*_alt_ = 64,2; CI_Bootstrap_ = (−17,08, −1,40); *t*(46) = −2,15, *p* = 0,02).

Hinsichtlich der Rollen bei der Arbeit besteht zum Teil Unklarheit, wie weit diese reichen. Arbeitsbezogene Rollenkonflikte werden in einem geringen Maß bzw. zum Teil wahrgenommen (Tab. 1). Dazu zählen unnötige Arbeitsaufgaben, heterogene Akzeptanzkriterien in Bezug auf die Ausführung von Tätigkeiten sowie widersprüchliche oder vom eigentlichen Standard abweichende Arbeitsvorgaben. Hier zeigt sich kein Altersunterschied in der Belegschaft (*p* = 0,26).

Die Ergebnisse zeigen weiterhin, dass die Mitarbeitenden in der Kommissionierung teilweise unsicher sind, ihren Arbeitsplatz zu verlieren (Tab. 1). Die Ängste bestehen dahingehend, arbeitslos zu werden, im Falle eines Jobverlustes keine neue Arbeit zu finden oder intern auf eine neue Arbeitsstelle versetzt zu werden. Die Sorge, dass der Arbeitsplatz durch den Einsatz neuer Technologien verloren geht, ist hingegen nur gering ausgeprägt. Hier gibt es keinen signifikanten Unterschied zu den anderen Unternehmen der Studie (*p* = 0,10) oder zwischen den Altersgruppen (*p* = 0,08) innerhalb des untersuchten Versandhändlers.

#### Herausforderungen

Die Beschäftigten in der Kommissionierung wurden auch bzgl. der Informationsbereitstellung zu Regelungen und Arbeitsabläufen befragt (Tab. 1). Die Ergebnisse verdeutlichen, dass die Kommissioniertätigkeit nur teilweise als vorhersehbar wahrgenommen wird. Dies betrifft die rechtzeitige Informierung über Veränderungen am Arbeitsplatz und die Verfügbarkeit von Informationen für eine reibungslose Erledigung der Arbeit. Innerhalb des Betriebes unterscheiden sich die Altersgruppen statistisch nicht signifikant (*p* = 0,56).

**Figure 6:**
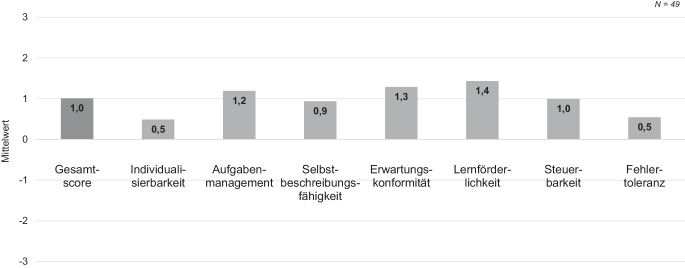

Als weitere Herausforderung wird die eingesetzte Kommissioniertechnik analysiert. Der Warenbegleitschein wurde hinsichtlich der Informationsdarstellung und Dialoggestaltung von den Befragten beurteilt (ISONORM 9241/110‑S, Prümper 1997). Dieser misst mittels bipolarer Items auf einer 7‑stufigen Skala, von sehr negativ (−−−) über unentschieden (−/+) bis sehr positiv (+++), sieben verschiedene Prinzipien menschlicher Informationsverarbeitung: Individualisierbarkeit, Aufgabenangemessenheit, Selbstbeschreibungsfähigkeit, Erwartungskonformität, Lernförderlichkeit, Steuerbarkeit und Fehlertoleranz (DIN EN ISO 9241). Die Ergebnisse deuten darauf hin, dass das verwendete Kommissionieretikett überwiegend als positiv eingeschätzt wurde (Abb. 6). In der Gesamtbewertung der aggregierten Skalen zeigt sich ein positiveres Bild der Kommissioniertechnik im Vergleich zu Techniken anderer Unternehmen (*M*_Betrieb_ = 1,0, *M*_Referenz_ = 0,6; CI_Bootstrap_ = (−0,10, 0,75); *t*(198) = 2,33, *p* = 0,01). Zwischen älteren und jungen Mitarbeiter/-innen des Unternehmens gibt es keinen Unterschied in der Bewertung der Kommissioniertechnik (*p* = 0,98).

### Ressourcen

In Anlehnung an das zuvor präsentierte Anforderungs-Ressourcen-Modell werden nun die Ergebnisse zum übergreifenden Bereich der Ressourcen dargestellt, sowohl deskriptiv als auch inferenzstatisch. Hierzu gehören sowohl Ressourcen, die während der Arbeit entstehen als auch im persönlichen Umfeld der Beschäftigten (Tab. 2).

**Table 2:** 

Skalen/Einzelitems	Herkunft	*N* Items	*M* ± SD	Sign. Unterschiede
*Arbeitsressourcen*				
Rollenklarheit	COPSOQ	4	71,9 ± 14,1	−;0
Einfluss bei der Arbeit	COPSOQ	4	24,1 ± 24	<;<
Entscheidungsspielraum	COPSOQ	4	32,7 ± 15,2	0;0
Entwicklungsmöglichkeiten	COPSOQ	4	33,8 ± 16,4	<;0
Bedeutung der Arbeit	COPSOQ	3	36,1 ± 16,4	<;0
Verbundenheit mit Arbeitsplatz (Commitment)	COPSOQ	4	51,5 ± 21,1	0;0
Soziale Unterstützung	COPSOQ	4	65,5 ± 21,5	0;0
Gemeinschaftsgefühl	COPSOQ	3	76,0 ± 20,3	0;0
Führungsqualität – Respekt	FBQ‑M	4	3,0 ± 0,9	>;0
Führungsqualität – Vertrauen	FBQ‑M	4	3,0 ± 1,1	<;>
Führungsqualität – Ermutigung	FBQ‑M	4	3,0 ± 1,0	0;0
Führungsqualität – Zuneigung	FBQ‑M	4	3,4 ± 1,0	>;0
Kommunikationsqualität	KQ	8	3,7 ± 0,8	>;0
*Persönliche Ressourcen*				
Work-(Family) Privacy-Konflikt	COPSOQ	5	45,2 ± 24,6	>;0

#### Arbeitsressourcen

Die Mitarbeitenden im Logistiklager des Versandhändlers berichten, dass sie nur in einem geringen Maß Einfluss auf Tätigkeitsinhalte und die Arbeitsmenge nehmen können oder mit wem sie zusammenarbeiten. Im Mittel ist dieser Wert auch geringer als in anderen Unternehmen (*M*_Betrieb_ = 24,1, *M*_Referenz_ = 38,5; CI_Bootstrap_ = (−22,40, −7,08); *t*(200) = −3,55, *p* < 0,001), die Altersgruppen innerhalb des Betriebs unterscheiden sich ebenfalls (*M*_jung_ = 14,7, *M*_alt_ = 29,5; CI_Bootstrap_ = (−26,13, −3,30); *t*(46) = −2,24, *p* < 0,01). Ein vergleichbares Ergebnis offenbart sich hinsichtlich der Entscheidungsbefugnisse der Beschäftigten. Es ist kaum möglich, Pausen‑, Arbeits- und Urlaubszeiten flexibel zu gestalten, was jedoch auch auf die Vergleichsunternehmen zutrifft. Zudem scheinen die Möglichkeiten persönlicher Entwicklung in der Kommissionierung im Durchschnitt begrenzt, d. h. eigeninitiativ oder kreativ zu handeln, sind nur eingeschränkt möglich. Diese Entwicklungsmöglichkeiten scheinen auch hinter denen anderer Unternehmen zu stehen (*M*_Betrieb_ = 33,8, *M*_Referenz_ = 49,4; CI_Bootstrap_ = (−21,57, −10,10); *t*(200) = −4,73, *p* < 0,001), sind jedoch in dem untersuchten Betrieb im Vergleich der Altersklassen gleich (*p* = 0,32).

Ein ähnliches Bild zeichnet sich in Bezug auf die wahrgenommene Bedeutung der Arbeitstätigkeit und der Verbundenheit mit dem Arbeitsplatz ab (Tab. 2). Die Beschäftigten bewerten ihre Arbeit nur teilweise als sinnvoll, wichtig und intrinsisch motivierend. Im Vergleich zu anderen Teilnehmern fällt hier der Mittelwert signifikant geringer aus (*M*_Betrieb_ = 36,1, *M*_Referenz_ = 49,32; CI_Bootstrap_ = (−18,92, −7,28); *t*(199) = −3,90, *p* = 0,001); innerhalb des untersuchten Betriebs gibt es gemäß der Altersgruppen keine Differenzierung (*p* = 0,56). Das Commitment ist ebenfalls nur zum Teil vorhanden. Die Beschäftigten geben an, nicht durchgängig stolz auf ihre Arbeit zu sein oder schätzen diese als wenig bedeutsam ein. Ein Unterschied zu anderen Unternehmen (*p* = 0,59) oder innerhalb der Belegschaftsaltersgruppen (*p* = 0,08) ist jedoch nicht statistisch signifikant.

In Bezug auf die Rollenklarheit bei der Erledigung von Arbeitsaufgaben wird ersichtlich, dass bei den Beschäftigten im Durchschnitt in hohem Maß Klarheit über Befugnisse, Arbeitsergebnisse, Arbeitsziele und den Verantwortungsbereich besteht. Auch hier gibt es keinen Unterschied zwischen jüngeren und älteren Beschäftigten (*p* = 0,18; Tab. 2).

Die Beschäftigten im dargestellten Unternehmen wurden gebeten, die gefühlte soziale Unterstützung von Kollegen und dem unmittelbaren Vorgesetzten sowie das erlebte Gemeinschaftsgefühl zu bewerten. Die Ergebnisse beider Skalen beschreiben ein positives Arbeitsklima (Tab. 2). Die Atmosphäre und Zusammenarbeit zwischen den Beschäftigten in der Belegschaft werden gleichermaßen als gut beschrieben. Bei Arbeitsproblemen erhalten die Mitarbeitenden sowohl von ihren Kollegen als auch von der Führungskraft häufig Hilfe und Unterstützung. Zwischen den Unternehmen unterscheiden sich weder die soziale Unterstützung (*p* = 0,56), noch das Gemeinschaftsgefühl (*p* = 0,26). Zwischen den beiden Altersgruppen innerhalb des Unternehmens gibt es ebenfalls keinen Unterschied in der sozialen Unterstützung (*p* = 0,42) oder bei dem Gemeinschaftsgefühl (*p* = 0,36).

Weiterhin wurde die Qualität der Beziehung zwischen den Beschäftigten und ihrem unmittelbaren Vorgesetzten (Wolfram und Mohr 2014) mit einem 5‑stufigen Antwortformat bewertet (1 = minimale Ausprägung = „trifft gar nicht zu“, 5 = maximale Ausprägung = „trifft völlig zu“). Die vier Skalen der Führungsqualität „Vertrauen“, „Respekt“, „Ermutigung“ und „Zuneigung“ liegen auf einem mittelmäßigen Niveau (Tab. 2). Im Fall von zwei Skalen liegen die Mittelwerte jedoch über denen konkurrierender Unternehmen, genauer bei „Respekt“ (*M*_Betrieb_ = 3,0, *M*_Referenz_ = 2,9; CI_Bootstrap_ = (−0,19, 0,40); *t*(199) = 0,69, *p* = 0,05) und „Zuneigung“ (*M*_Betrieb_ = 3,4, *M*_Referenz_ = 3,1; CI_Bootstrap_ = (−0,05, 0,63); *t*(197) = 1,66, *p* = 0,09). Nur das „Vertrauen“ ist geringer ausgeprägt als in anderen Unternehmen (*M*_Betrieb_ = 3,04, *M*_Referenz_ = 3,36; CI_Bootstrap_ = (−0,63, 0,01); *t*(198) = −2,06, *p* = 0,05). Lediglich die Skala „Ermutigung“ zeigt keinen signifikanten Unterschied (*p* = 0,74). Im Altersvergleich zeigt sich bei der Skala „Vertrauen“ ein signifikant höherer Wert bei jüngeren Mitarbeitern (*M*_jung_ = 3,3, *M*_alt_ = 2,8; CI_Bootstrap_ = (−0,09, 1,09); *t*(45) = 1,56, *p* = 0,10). Bei den Kategorien „Respekt“ (*p* = 0,93), „Ermutigung“ (*p* = 0,60) und „Zuneigung“ (*p* = 0,87) zeigen sich keine Effekte.

Des Weiteren wurde erfasst, wie die Mitarbeitenden der Kommissionierung bei dem untersuchten Versandhändler die Kommunikationsqualität zur Führungskraft wahrnehmen (Mohr et al. 2014). Hierbei wurden beide Richtungen der Kommunikation das Ausmaß berücksichtigt (1 = minimale Ausprägung = „trifft gar nicht zu“, 5 = maximale Ausprägung = „trifft völlig zu“, Mohr et al. 2014). Die Ergebnisse geben Hinweise darauf, dass die Qualität der Kommunikation überwiegend positiv wahrgenommen wird, bspw. gibt die Führungskraft klare und verständliche Anweisungen, lässt Mitarbeitende ausreden und liefert ausführliche Informationen (Tab. 2). Der Mittelwert dieser Skala ist im Vergleich zu anderen Unternehmen signifikant höher, was die deskriptiven Ergebnisse zusätzlich bestärkt (*M*_Betrieb_ = 3,7, *M*_Referenz_ = 3,5; CI_Bootstrap_ = (−0,02, 0,51); *t*(200) = 1,66, *p* = 0,08). Einen Unterschied zwischen den Altersgruppen gibt es nicht (*p* = 0,57; Tab. 2).

#### Persönliche Ressourcen

Die Mehrheit der Arbeitenden in der Kommissionierung weist einen Hauptschulabschluss bzw. Volksschulabschluss (51 %) oder einen Realschulabschluss, Mittlere Reife bzw. Abschluss Polytechnische Oberschule (POS) (30,7 %) auf (gegenüber keinem Abschluss bzw. sonstigem Schulabschluss: 10,2 %; allgemeine bzw. fachgebundene Hochschulreife, Abitur, Fachhochschulreife: 8,1 %). Hinsichtlich des beruflichen Ausbildungsabschlusses ergibt sich folgendes Bild: ohne bzw. anderer Ausbildungsabschluss: 31,9 %; beruflich-schulischer bzw. beruflich-betrieblicher Ausbildungsabschluss: 61,7 %; Fach‑, Meister‑, Technikerschule bzw. Fach‑/Hochschulabschluss: 6,4 %. Um einen Vergleich des hier näher beschriebenen Versandhändlers und den übrigen Unternehmen zu ermöglichen, wurden die Referenzbetriebe (*N*_Referenz_ = 158) ebenfalls auf das Bildungsniveau geprüft. Dieses liegt auf einem vergleichbaren Niveau (Hauptschule/Volkshochschule: 40 %, Realschule/mittlere Reife: 30 %, polytechnische Oberschule: 3 %, Hochschul- & Fachhochschulreife: 18 %, keinen Schulabschluss: 6 %, sonstiger Abschluss: 3 %). Lediglich das Geschlechterverhältnis (Weiblich_Referenz_ = 34 %) weicht stark vom untersuchten Betrieb ab.

Bezogen auf die Vereinbarkeit von Privat- und Familienleben mit dem Beruf wurde der Work-(Family) Privacy-Konflikt (WFK) verwendet (Netemeyer et al. 1996). Dieser beschreibt eine teilweise Unvereinbarkeit von familiären und beruflichen Rollen, genauer gesagt wird beurteilt, in welchem Ausmaß die Arbeit das Privat- und Familienleben stört. Deskriptive Analysen verdeutlichen, dass das private und familiäre Leben der befragten Mitarbeitenden in der Kommissionierung im Durchschnitt gelegentlich vom Arbeitsleben gestört wird (Tab. 2). Zeitbasierte Konflikte, dass private oder familiäre Pläne und Aktivitäten aufgrund beruflicher Verpflichtungen geändert werden müssen, treten dabei am häufigsten auf. Daneben werden manchmal belastungsbasierte Konflikte erlebt, z. B. dass der mit der Arbeit verbundene Stress es schwierig macht, Verpflichtungen des Privat- und Familienlebens nachzukommen. Der Work-Family-Konflikt scheint in diesem Unternehmen stärker auszufallen als in den übrigen untersuchten Unternehmen (*M*_Betrieb_ = 45,2, *M*_Referenz_ = 34,1; CI_Bootstrap_ = (3,11, 19,10); *t*(203) 2,64, *p* < 0,01); es findet sich jedoch kein Unterschied zwischen den Altersgruppen (*p* = 0,17).

Des Weiteren wurden die Beschäftigten in der Kommissionierung nach ihrer Absicht, den Arbeitsplatz zu wechseln, gefragt. Es wurde erhoben, wie häufig die Mitarbeitenden im Laufe der letzten 12 Monate daran gedacht haben, den Beruf aufzugeben (1 = minimale Ausprägung = „nie“, 5 = maximale Ausprägung = „jeden Tag“) (Hasselhorn und Müller 2004). Zwar haben 60,4 % noch nie derartige Gedanken gehabt, jedoch denken fast 29,6 % der Befragten mehrmals im Jahr und immerhin 10,4 % einige Male im Monat bis täglich darüber nach.

### Zielgrößen „Arbeitsfähigkeit, Arbeitszufriedenheit und Gesundheit“

Deskriptive und inferenzstatistische Ergebnisse der Zielgrößen Arbeitsfähigkeit, Arbeitszufriedenheit und Gesundheit sind in Tab. 3 dargestellt.

**Table 3:** 

Skalen/Einzelitems	Herkunft	*N* Items	*M* ± SD	Sign. Unterschiede
*Arbeitsfähigkeit*				
Derzeitige Arbeitsfähigkeit im Vergleich zu der besten je erreichten Arbeitsfähigkeit	WAI	1	7,4 ± 2,42	<;>
Arbeitsfähigkeit im Vergleich zu den Anforderungen der Arbeitstätigkeit	WAI	2	7,3 ± 1,4	<;0
*Arbeitszufriedenheit*	COPSOQ	7	59,2 ± 12,9	0;0
*Gesundheit*				
Allgemeiner Gesundheitszustand	COPSOQ	1	6,1 ± 2,2	<;>
Verhaltensbezogene Stresssymptome	COPSOQ	8	4,8 ± 21,6	0;0
Kognitive Stresssymptome	COPSOQ	4	33,6 ± 17,9	0;0
Körperliche Beschwerden	PSB	20	2,5 ± 0,9	>;<
Regenerationsbedürfnis	NFR	11	3,1 ± 1,9	>;0

#### Arbeitsfähigkeit

Die derzeitige subjektive Arbeitsfähigkeit im Vergleich zu der besten, je erreichten Arbeitsfähigkeit wurde mit einem 11-stufigen Einzelitem erfragt (0 = minimale Ausprägung = „völlig arbeitsunfähig“, 10 = maximale Ausprägung = „derzeit die beste Arbeitsfähigkeit“, Tuomi et al. 1998). Der Durchschnittswert der Arbeitsfähigkeit der Mitarbeitenden in der Kommissionierung liegt im positiven Bereich (Tab. 3). Im Vergleich zu anderen Unternehmen fällt dieser Aspekt der Arbeitsfähigkeit geringer, jedoch nicht signifikant aus (*p* = 0,83). Jüngere Mitarbeiter/-innen in der Kommissionierung zeigen dabei wie erwartet einen höheren allerdings nicht signifikanten Durchschnittswert als ältere (*p* = 0,28). Die gewichtete Berechnung der Arbeitsfähigkeit, im Detail die Arbeitsfähigkeit im Vergleich zu den körperlichen und psychischen Anforderungen der Arbeitstätigkeit, liegt auf einem vergleichbar hohen Niveau (Tab. 3). Jedoch fällt die Einschätzung der Mitarbeitenden in diesem Aspekt geringer aus als in anderen Unternehmen (*M*_Betrieb_ = 7,3, *M*_Referenz_ = 7,7; CI_Bootstrap_ = (−0,86, 0,04); *t*(199) = −1,64, *p* = 0,09) ohne eine Differenzierung der jüngeren und älteren Belegschaft im untersuchten Betrieb (*p* = 0,09).

#### Arbeitszufriedenheit

Die Arbeitszufriedenheit wurde auf einem 4‑stufigen Antwortformat gemessen (0 = minimale Ausprägung = „sehr unzufrieden“, 100 = maximale Ausprägung = „sehr zufrieden“; COPSOQ, Nübling et al. 2005). Im Durchschnitt liegt die Arbeitszufriedenheit auf einem moderaten Niveau (Tab. 3). Im Detail zeigt sich, dass die Zufriedenheit der Mitarbeitenden hinsichtlich der Arbeitskolleg/-innen am größten ist. Die restlichen Aspekte der Arbeitssituation liegen im mittleren Wertebereich des Kontinuums zwischen „sehr unzufrieden“ und „sehr zufrieden“, wie die Berufsperspektiven, der Führungsstil, die Herausforderungen und Fertigkeiten, die mit der Arbeit einhergehen oder die persönlichen Fähigkeiten, die für die Arbeit genutzt werden können. Der geringste Zufriedenheitswert wird dabei bei den körperlichen Anforderungen erzielt. Hier gibt es weder signifikante Unterschiede zwischen dem hier betrachteten und den übrigen Unternehmen (*p* = 0,25), noch zwischen jüngeren und älteren Mitarbeiter/-innen des untersuchten Versandhändlers (*p* = 0,74).

#### Gesundheit

Tab. 3 stellt dar, dass die Mitarbeitenden in der Kommissionierung ihren allgemeinen Gesundheitszustand auf einem moderaten Niveau mit der Tendenz zu einer positiven Ausprägung bewerteten (0 = minimale Ausprägung = „schlechtester denkbarer Gesundheitszustand“, 10 = maximale Ausprägung = „bester denkbarer Gesundheitszustand“; COPSOQ, Nübling et al. 2005). Dieser Wert ist im Vergleich zu anderen Unternehmen signifikant geringer (*M*_Betrieb_ = 6,1, *M*_Referenz_ = 7,2; CI_Bootstrap_ = (−1,79, −0,36); *t*(203) = −3,14, *p* < 0,01) und erwartungskonform unter der älteren Belegschaft geringer ausgeprägt (*M*_jung_ = 7,7, *M*_alt_ = 5,1; CI_Bootstrap_ = (1,52, 3,56); *t*(46) = 4,74, *p* < 0,001).

Hinsichtlich der Wirkungen von Fehlbeanspruchungen weisen die Mitarbeitenden in der Kommissionierung im Durchschnitt kaum Stresssymptome auf (Tab. 3). Hierbei sind kognitive von verhaltensbezogenen Wirkungen zu unterscheiden. Bei den verhaltensbezogenen Stresssymptomen wird etwas häufiger angegeben, dass die Zeit fehlte, sich zu entspannen oder dass es schwerfiel, glücklich zu sein. Bei den kognitiven Stresssymptomen zeichnet sich ein ähnliches Bild ab: auch sie treten im Durchschnitt kaum bis etwas auf. Es wurde am häufigsten angegeben, dass es etwas schwerfiel, sich bei der Arbeit zu konzentrieren. Beide Maße unterscheiden sich nicht signifikant zwischen dem dargestellten und den übrigen untersuchten Betrieben, weder kognitiv (*M*_Betrieb_ = 33,5, *M*_Referenz_ = 26,0; CI_Bootstrap_ = (1,28, 13,23); *t*(201) = 2,12, *p* = 0,01), noch im Verhalten (*M*_Betrieb_ = 34,8, *M*_Referenz_ = 28,6; CI_Bootstrap_ = (−0,58, 13,20); *t*(200) = 1,60, *p* = 0,08). Auch innerhalb der Belegschaft gibt es keine altersspezifischen Unterschiede in kognitiven (*M*_jung_ = 27,8, *M*_alt_ = 37,1; CI_Bootstrap_ = (−18,70, 0,180); *t*(46) = −1,80, *p* = 0,05) oder verhaltensbezogenen (*M*_jung_ = 29,6, *M*_alt_ = 38,7; CI_Bootstrap_ = (−20,45, 1,45); *t*(45) = −1,43, *p* = 0,12) Auswirkungen. Ein vergleichbares Ergebnis konnte hinsichtlich des Regenerationsbedürfnis (0 = minimale Ausprägung, 11 = maximale Ausprägung; van Veldhoven und Broersen 2003) erzielt werden (Tab. 3): Die gefühlte Ermüdung ist bei den Beschäftigten in der Kommissionierung gering ausprägt, jedoch nicht so gering wie in anderen Unternehmen (*M*_Betrieb_ = 3,1, *M*_Referenz_ = 2,3; CI_Bootstrap_ = (0,10, 1,37); *t*(205) = 2,05, *p* = 0,03). Zwischen den Altersgruppen gibt es keinen Unterschied (*p* = 0,16).

**Figure 7:**
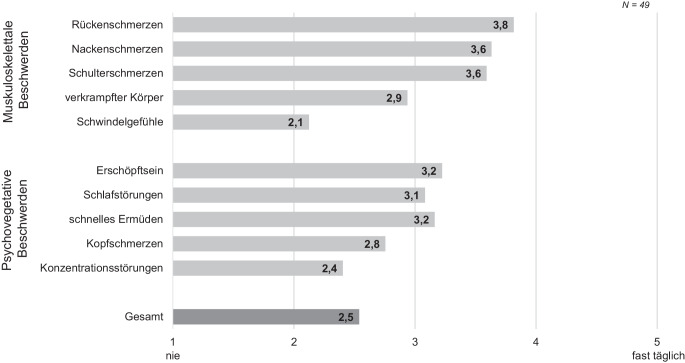

Außerdem wurde die Häufigkeit verschiedener körperlicher Beschwerden erfragt (1 = minimale Ausprägung = „nie“, 5 = maximale Ausprägung = „fast täglich“; Mohr und Müller 2014; Tab. 3). Die deskriptiven Ergebnisse verdeutlichen, dass muskuloskelettale Beschwerden, gemessen am Durchschnittswert der Beschwerden, häufiger auftreten (Abb. 7). Dazu zählen Rücken‑, Nacken- sowie Schulterschmerzen sowie ein verkrampfter Körper. Daneben kommen psychovegetative Beschwerden überdurchschnittlich häufig vor, wie Erschöpfung, schnelles Ermüden, Schlafstörungen oder Kopfschmerzen. Diese körperlichen Beschwerden sind im Vergleich zu anderen Unternehmen signifikant stärker ausgeprägt (*M*_Betrieb_ = 2,5, *M*_Referenz_ = 2,0; CI_Bootstrap_ = (0,32, 0,86); *t*(203) = 4,88, *p* < 0,001). Wie erwartet sind die Beschwerden bei älteren Mitarbeitern stärker ausgeprägt als bei den jüngeren Beschäftigten (*M*_jung_ = 2,2, *M*_alt_ = 2,8; CI_Bootstrap_ = (−1,02, −0,12); *t*(46) = −2,35, *p* = 0,01).

## Diskussion

Ziel der vorliegenden Studie war es, die Arbeitssituation in der Kommissionierung unterschiedlicher Betriebe zu beurteilen. Vor dem Hintergrund eines stark anwachsenden E‑Commerce-Bereichs wurden die Befragungsergebnisse eines großen Versandhändlers exemplarisch beschrieben und mit anderen an der Feldstudie teilgenommenen Betrieben verglichen. Die Arbeitssituation wurde hinsichtlich der Anforderungen und Ressourcen beurteilt. Als theoretischer Rahmen diente das JD-R-Modell und als Messinstrument wurden bewährte Skalen aus der Arbeitswissenschaft verwendet. Auffällig in dem untersuchten Betrieb ist der hohe Anteil weiblicher Mitarbeiter (88 %) im Vergleich zu den Referenzbetrieben (34 %). Der Anteil älterer Mitarbeiter ist hoch und liegt bei ca. 49 Jahren (75 % der Beschäftigten sind 45 Jahre oder älter).

In Bezug auf die Anforderungen zeigt sich, dass vor allem Stressoren wie sensorische und quantitative Anforderungen als sehr hoch bewertet werden. Insbesondere die hohe Bewertung von manuellem und visuellem Arbeiten, Daueraufmerksamkeit und die damit verbundene hohe Konzentrationsleistung legen nahe, dass diese Stressoren stark beanspruchend wirken, wobei sich interessanterweise hier kein Altersunterschied zeigt. In der kognitiven Domäne spielen insbesondere attentionales Multitasking und Verantwortungsübernahme eine starke Rolle, die von den älteren Mitarbeitern als beanspruchender bewertet werden. Quantitative Stressoren, bedingt durch schnelle Arbeitsweise und Überstunden, werden von jungen und älteren MitarbeiterInnen als gleichermaßen beanspruchend bewertet. In Bezug auf die quantitativen Stressoren zeigt sich zumindest ein Trend dahin, dass hier die älteren MitarbeiterInnen die Beanspruchung höher bewerten. Im Vergleich zu den übrigen Unternehmen der Befragung findet sich eine höhere sensorische, jedoch eine geringere kognitive Beanspruchung, was eventuell mit der Nutzung der eher analogen, einfacheren Kommissioniertechnik in Zusammenhang steht. Weiterhin zeigt sich, dass sich etwa die Hälfte der Mitarbeiter durch die Schichtarbeit belastet fühlt und Sorgen und Ängste bezüglich des Arbeitsplatzverlusts nicht zu vernachlässigen sind. Das Niveau in der Bewertung der Arbeitsplatzunsicherheit unterscheidet sich nicht zwischen dem untersuchten Unternehmen und den Referenzbetrieben.

Als Herausforderung werden in unserer Studie Technik und Vorhersehbarkeit der Arbeit eingeordnet. Die Analysen zeigen, dass der Warenbegleitschein als genutzte Kommissioniertechnik insgesamt als positiv empfunden und auch signifikant positiver als die Methoden der übrigen Unternehmen bewertet wird. Die Vorhersehbarkeit der Arbeit ist jedoch nicht sonderlich stark ausgeprägt, was diese Herausforderung eher zu einem Stressor als zu einer Ressource werden lässt und offensichtlich durch eine verbesserte Informationsinfrastruktur ausgeglichen werden könnte.

Bezüglich der Arbeitsressourcen scheinen vor allem Ressourcen wie Gemeinschaftsgefühl, Kommunikationsqualität, Führungsqualität, Rollenklarheit und soziale Unterstützung eine bedeutende Rolle in der Bewertung der Arbeitssituation zu spielen. Im Vergleich zu den anderen Unternehmen wird insbesondere das Verhältnis zu Führungspersonen sehr positiv bewertet und kann somit als bedeutende Arbeitsressource für das untersuchte Unternehmen gesehen werden. Die Verbundenheit zum Arbeitsplatz wird auf einem eher mittleren Niveau bewertet. Eher niedrig werden die Bedeutung der Arbeit, der Entscheidungsspielraum, die Entwicklungsmöglichkeiten und der Einfluss bei der Arbeit bewertet. Darüber hinaus werden die Bedeutung der Arbeit, die Entwicklungsmöglichkeiten und der Arbeitseinfluss im untersuchten Unternehmen deutlich niedriger bewertet als in den Vergleichsunternehmen. Bezüglich der zuletzt genannten Ressourcen, besteht hier demnach eher ein Defizit.

Als persönliche Ressourcen werden in unserer Studie die Work-Life-Balance, Bildung und die Absicht, den Arbeitsplatz zu wechseln, gesehen. Hier zeigt sich, dass der Einfluss auf das Privatleben auf einem mittleren Niveau bewertet wird und somit nicht zu vernachlässigen ist und zudem nur bedingt als persönliche Ressource gewertet werden kann. Darüber hinaus wird der Einfluss auf das Privatleben durch berufliche Verpflichtungen im dargestellten Betrieb deutlich höher bewertet als in den Vergleichsunternehmen. Die Absicht, den Arbeitsplatz zu wechseln, scheint eher eine geringe Rolle zu spielen. Die Wechselabsicht wird in dieser Feldstudie als Ressource der persönlichen Weiterentwicklung gesehen. Studien geben Hinweise darauf, dass die Wahrscheinlichkeit des Verbleibs am Arbeitsplatz u. a. durch die persönliche Qualifizierung oder die berufliche Weiterentwicklung bedingt wird (Taylor und Oetzel 2020; Winters 2019).

Bezüglich der „Zielvariablen“ ist anzumerken, dass die Arbeitszufriedenheit auf einem mittleren Niveau liegt und sich von den Referenzunternehmen nicht unterscheidet. Die Bewertung des allgemeinen Gesundheitszustandes liegt etwas über dem mittleren Niveau, jedoch statistisch signifikant unter dem Mittelwert der anderen Unternehmen und ist innerhalb der Belegschaft bei den älteren Beschäftigten geringer. Im Hinblick auf verhaltensbezogene und kognitive Stresssymptome, die im unteren Drittel der Skala bewertet werden, gibt es keine Unterschiede zwischen den Unternehmen oder innerhalb der Belegschaft. Körperliche Beschwerden werden ebenfalls als eher niedrig beurteilt (unteres Skalenviertel) sind jedoch in dem dargestellten Betrieb signifikant höher als in den Vergleichsbetrieben. Ebenfalls zeigt sich, dass die älteren Beschäftigten die körperlichen Beschwerden etwas höher beurteilen als die jüngeren. Daneben wird auch das Regenerationsbedürfnis eher im Bereich des unters Skalendrittels angegeben und ist höher als in den Vergleichsunternehmen.

## Implikationen

Versucht man vor dem Hintergrund des JD-R-Modells eine eher deskriptive Bilanz zwischen Anforderungen und Ressourcen zu ziehen, so halten sich in dem dargestellten Betrieb offensichtlich Stressoren und Arbeitsressourcen in etwa die Waage. Bei einem numerischen Vergleich der Skalen, werden die Stressoren auf einem Prozentrang von 57 % und die Arbeitsressourcen auf einem Rang von 56 % bewertet. Bezüglich der dominanten Stressoren (sensorische, kognitive und quantitative Anforderungen) könnte eine Verbesserung der Ressourcen bezüglich Autonomie (Einfluss bei der Arbeit, Entscheidungsspielraum) hilfreich sein. Die zeitliche und fachliche Arbeitsüberforderung, Rollenprobleme und ein Mangel an Kontrolle/Autonomie werden in der Literatur als signifikante Korrelate für längerfristige, negative Auswirkungen auf die Arbeitsfähigkeit identifiziert (Alarcon 2011).

Die als überwiegend positiv bewerteten Ressourcen, wie Gemeinschaftsgefühl, Kommunikationsqualität, Führungsqualität, Rollenklarheit und soziale Unterstützung sollten weiterhin gestärkt und ausgebaut werden. Frühere Studien (Xanthopoulou und Bakker 2007) haben gezeigt, dass verschiedene Arbeitsressourcen, wie soziale Unterstützung, Coaching durch den Vorgesetzten, Leistungsfeedback und Möglichkeiten zur beruflichen Weiterentwicklung, positiv mit dem Engagement am Arbeitsplatz zusammenhängen. Engagierte Mitarbeiter haben wiederum ein hohes Maß an Energie, sind von ihrer Arbeit begeistert und gehen oft völlig in ihrer Arbeit auf (Macey und Schneider 2008; May et al. 2004). Darüber hinaus wirkt sich das Engagement positiv auf Kundenzufriedenheit (Salanova et al. 2005) und finanziellen Ertrag (Xanthopoulou et al. 2009) aus.

Analog zu Certa und Schröder (2020) kommen wir zu dem Schluss, dass sich die Arbeitsfähigkeit der Beschäftigten offensichtlich bereits mit Hilfe scheinbar „weicher“ Maßnahmen verbessern lässt, z. B. durch die Stärkung sozialer Unterstützung unter Kollegen und Führungskräfte sowie ein Betriebsklima, das sowohl auf Anerkennung und Gleichheit als auch auf Rollenklarheit basiert. Führungskräfte können hierbei als Moderatoren und Gestalter eine besondere Rolle spielen.

Wie oben erwähnt können Arbeitsplatzanforderungen, vor allem in Form eines starken Termin- und Leistungsdrucks, als äußerst bedeutend für die Arbeitsfähigkeit angesehen werden. Certa und Schröder (2020) schlagen diesbezüglich vor, den Arbeitsfluss zu verbessern ohne die Intensität zu erhöhen. Solche Maßnahmen können prinzipiell durch den Einsatz von geeigneten Kommissioniertechnologien erreicht werden (Glock et al. 2020). In unserer Studie wird der eingesetzte Warenbegleitschein bezüglich seiner Gebrauchstauglichkeit etwas besser als der Durchschnitt bewertet. Fortschrittliche Technologien, wie beispielsweise mobile Datenendgeräte, Pick-by-Voice- oder Pick-by-Vision-Lösungen, haben grundsätzlich das Potenzial, Lagermitarbeiter in Bezug auf sensorische und kognitive Anforderungen zu unterstützen (z. B. Baechler et al. 2016). Assistive Technologien sollten dabei so gestaltet sein, dass sie bezüglich der Stressor-Ressourcenbilanz auf der Seite der Ressourcen angesiedelt werden können (Demerouti 2020) und die Handlungsautonomie nicht beschränken, sondern stärken (Certa und Schröder 2020). Generell ist es sinnvoll, die eingesetzte Kommissioniertechnik entsprechend der individuellen Situation so auszuwählen. Ein gewohnte, analoge Kommissioniertechnik, wie der Warenbegleitschein, kann jedoch Vorteile haben, wie z. B. eine hohe Individualisierbarkeit, und somit besser als digitale, Kommissioniertechniken abschneiden. Das oft allgemeine Verständnis, dass neue Technologien vorzuziehen sind, da sie einen Fortschritt mit sich bringen, wäre zu prüfen.

Zunächst bedeutet die Einführung von neuen Technologien im Unternehmen immer, dass ein Veränderungsprozess durchlebt werden muss. Damit dieser erfolgreich ist, muss er von den Beschäftigten vertretbar sein. Eine Studie von Elbert et al. (2017) zeigt, dass die Einführung eines Assistenzsystems zur Lagernavigation oft nicht zielführend eingesetzt werden konnte. Kommissionierer verfolgten weiter ihre gewohnten Routen und wichen von den optimalen Routen, die das System vorschlug, ab. Ein Grund hierfür ist möglicher Weise der Eingriff in die Entscheidungsfreiheit der Kommissionierer.

Die digitale Transformation benötigt zudem den Aufbau neuer Fähigkeiten der Beschäftigten (Sousa und Rocha 2019). Hier sind sowohl jüngere als auch ältere Beschäftigte zu berücksichtigen und bedarfsgerechte Angebote zur Verfügung zu stellen. Die digitale Transformation bietet hier grundsätzlich den Beschäftigten die Möglichkeit, Einfluss auf die Arbeit zu haben und den eigenen Entscheidungsspielraum zu erweitern. Eine ergonomische Gestaltung der Mensch-Maschine Schnittstelle ist zusätzlich relevant für einen zielführenden Einsatz der Kommissioniertechnik. In dieser Studie zeigt sich bspw., dass die Schriftgröße des Kommissioniertetiketts zu klein war. Klumpp et al. (2019) machen deutlich, dass die größte Herausforderung darin besteht, die Mensch-Technik Interaktion so zu gestalten, dass Beschäftigte und automatisierte Systeme in der Logistik effektiv kollaborieren. Die Autoren sehen die Akzeptanz der Beschäftigten, die Intuition, die Mensch-Maschine-Interaktion sowie Konzepte wie die Selbstwirksamkeit, als zentrale Faktoren für die zukünftige Wettbewerbsfähigkeit von Logistik-Unternehmen.

## Limitationen und Ausblick

Bei dieser Studie handelt es sich um ein rein querschnittliches Design und aufgrund der niedrigen Anzahl an Teilnehmern und eher heterogenen Stichproben in dem dargestellten Unternehmen und den Vergleichsunternehmen war es nicht möglich, Regressionsanalysen durchzuführen. Daher erfolgte die Analyse der Arbeitssituation ausschließlich anhand bewährter Bewertungsskalen aus der Arbeitswissenschaft. Bei zukünftig größeren Stichproben sollen entsprechende Methoden verwendet werden, um kausale Zusammenhänge zwischen insbesondere psychischen Stressoren, Herausforderungen und Ressourcen mit Zielgrößen wie Arbeitszufriedenheit, Arbeitsfähigkeit und Gesundheit detaillierter aufklären zu können. Auch waren verwendete technische Hilfsmittel und Methoden in der Kommissionierung der Vergleichsunternehmen so heterogen, dass ein Vergleich den Rahmen dieses Beitrags sprengen würde.

Generell hat sich gezeigt, dass sich die Datenerhebung innerhalb der Unternehmen oft schwierig gestaltet und häufig nur ein Teil der Belegschaft an den Befragungen teilnimmt. Einschränkend bezüglich der Generalisierbarkeit der Stichprobe (z. B. auf andere Unternehmen aus der E‑Commerce-Branche) muss daher erwähnt werden, dass die Stammbelegschaft nur zum Teil in die Befragung aufgenommen werden konnte. Gründe dafür waren Unabkömmlichkeit oder Sprachbarrieren bezüglich der schriftlichen Befragung. Zukünftig soll die Gesamtstichprobe durch Befragungen in weiteren Logistikunternehmen erweitert werden, um die Arbeitssituation insbesondere in der Intralogistik weiter analysieren und unterschiedliche Typen von Unternehmen vergleichen zu können. Unseres Erachtens besteht aufgrund der spezifischen Arbeitsanforderungen im Lagerbereich und dem zunehmenden Zwang der Digitalisierung ein besonderer Bedarf Gesundheit, Arbeitsfähigkeit und Wohlbefinden in diesem bisher eher vernachlässigten Arbeitsbereich besser aufzuklären, um die Arbeit auch im Kontext von Industrie 4.0 human gestalten zu können.
